# Novel integrase mutations linked to genotypic DTG resistance in African non-B HIV-1 strains: the DTG RESIST study

**DOI:** 10.1093/jac/dkag088

**Published:** 2026-03-24

**Authors:** Nuri Han, Tom Loosli, Mamatha Sauermann, İpek Çelikağ, Nanina Anderegg, Bertha C Baye, Carolyn Bolton Moore, Lydia Buzaalirwa, Helen Byakwaga, Cleophas Chimbetete, Peter V Ebasone, Suzanne Goodrich, Jacqueline Huwa, Charles Kasozi, Adolphe Mafoua, Arcel Christ Massamba, Eugène Messou, Albert Minga, Gad Murenzi, Guy Muula, Winnie Muyindike, Shirelle J Naidoo, Dominique Mahambou Nsonde, Armel G Poda, Richard Ramdé, Aggrey Semeere, Lavanya Singh, Huldrych F Günthard, Matthias Egger, Jennifer Giandhari, Richard Lessells, Roger D Kouyos

**Affiliations:** Departmentof Infectious Diseases and Hospital Epidemiology, University Hospital Zurich, University of Zurich, Zurich, Switzerland; Institute of Medical Virology, University of Zurich, Zurich, Switzerland; Departmentof Infectious Diseases and Hospital Epidemiology, University Hospital Zurich, University of Zurich, Zurich, Switzerland; Institute of Medical Virology, University of Zurich, Zurich, Switzerland; Institute of Social and Preventive Medicine, University of Bern, Bern, Switzerland; Institute of Social and Preventive Medicine, University of Bern, Bern, Switzerland; Institute of Social and Preventive Medicine, University of Bern, Bern, Switzerland; KwaZulu-Natal Research Innovation and Sequencing Platform, University of KwaZulu-Natal, Durban, South Africa; Centre for Infectious Disease Research in Zambia, Lusaka, Zambia; School of Medicine, University of Alabama at Birmingham, Birmingham, AL, USA; Medical Department, AIDS Healthcare Foundation Uganda Cares, Masaka, Uganda; Faculty of Medicine, Mbarara University of Science and Technology, Mbarara, Uganda; Research and Training Unit, Newlands Clinic, Harare, Zimbabwe; Department of Communicable Diseases, Clinical Research Education Networking and Consultancy (CRENC), Yaoundé, Centre Region, Cameroon; Division of Infectious Diseases, Indiana University School of Medicine, Indianapolis, IN, USA; Lighthouse Trust, Lilongwe, Malawi; Public Health Department, Regional Referral Hospital, Masaka, Uganda; Centre de Traitement Ambulatoire, Pointe Noire, Republic of the Congo; Centre de Traitement Ambulatoire, Brazzaville, Republic of the Congo; Centre de Prise en Charge, de Recherche et de Formation, Abidjan, Côte d'Ivoire; Programme PAC-CI, Abidjan, Côte d'Ivoire; Department of Research, Goodlife Access and Research for Development, Kigali, Rwanda; Centre for Infectious Disease Research in Zambia, Lusaka, Zambia; Faculty of Medicine, Mbarara University of Science and Technology, Mbarara, Uganda; KwaZulu-Natal Research Innovation and Sequencing Platform, University of KwaZulu-Natal, Durban, South Africa; Centre de Traitement Ambulatoire, Brazzaville, Republic of the Congo; Equipe de Recherche Clinique, Laboratoire des Pathogènes Emergents et Reémergents, Université Nazi BONI, Bobo- Dioulasso, Burkina Faso; Service des Maladies Infectieuses, Hôpital de Jour, Centre Hospitalier Universitaire Sourô SANOU, Bobo-Dioulasso, Burkina Faso; Department of Infectious Diseases, Souro Sanou University Hospital, Bobo-Dioulasso, Burkina Faso; Infectious Diseases Institute, Makerere University, Kampala, Uganda; KwaZulu-Natal Research Innovation and Sequencing Platform, University of KwaZulu-Natal, Durban, South Africa; Departmentof Infectious Diseases and Hospital Epidemiology, University Hospital Zurich, University of Zurich, Zurich, Switzerland; Institute of Medical Virology, University of Zurich, Zurich, Switzerland; Departmentof Infectious Diseases and Hospital Epidemiology, University Hospital Zurich, University of Zurich, Zurich, Switzerland; Centre for Infectious Disease Epidemiology and Research, Faculty of Health Sciences, University of Cape Town, Cape Town, South Africa; Population Health Sciences, Bristol Medical School, University of Bristol, Bristol, UK; KwaZulu-Natal Research Innovation and Sequencing Platform, University of KwaZulu-Natal, Durban, South Africa; KwaZulu-Natal Research Innovation and Sequencing Platform, University of KwaZulu-Natal, Durban, South Africa; Centre for the AIDS Programme of Research in South Africa, Durban, South Africa; Departmentof Infectious Diseases and Hospital Epidemiology, University Hospital Zurich, University of Zurich, Zurich, Switzerland; Institute of Medical Virology, University of Zurich, Zurich, Switzerland

## Abstract

**Background:**

Integrase mutations associated with dolutegravir resistance have been well characterized, but based on limited data from non-B subtypes.

**Objectives:**

We aim to identify potential integrase mutations not currently classified as integrase strand transfer inhibitor (INSTI) resistance mutations (DRMs) in individuals with viremia on dolutegravir-based regimens.

**Methods:**

We included integrase sequences from DTG RESIST study sites in African countries. These were interpreted using Stanford HIVdb v9.8. We used a viral genome-wide association study-like approach restricted to the integrase region (INT-WAS) to identify mutations not classified as major or accessory INSTI DRMs but occurring more frequently in sequences carrying major INSTI DRMs than in those without major INSTI DRMs. We performed the same INT-WAS analysis with drug-naïve sequences from the Los Alamos HIV-1 database to test whether these identified mutations were enriched among sequences from individuals with viraemia whilst receiving DTG-based treatment.

**Results:**

Among 382 sequences, 104 (27.2%) showed at least intermediate dolutegravir resistance. Twelve integrase mutations not classified as major or accessory DRMs (S39R, L45I, I72L, L74I, V79I, I113V, S119R, K156N, I208M, T218M, A265V, and R284G) were significantly associated with predicted DTG resistance. Among them, V79I [adjusted odds ratio (aOR) 167.1, 95% credible interval (CrI) 17.9–2947.6] and I72L (aOR 65.6, 95% CrI 6.6–1273.7) were strongly associated. S39R, L45I, V79I, S119R, and K156N were linked to established INSTI resistance pathways, and I72L, L74I, V79I, K156N, I208M, and R284G were overrepresented in sequences from viraemic individuals on DTG-based treatment relative to drug-naïve sequences.

**Conclusions:**

We identified several amino acid substitutions outside the established DRMs that are strongly associated with predicted dolutegravir resistance. Dolutegravir resistance evolution is complex and likely involves mutations not currently classified as DRMs.

## Introduction

Dolutegravir (DTG) is a second-generation integrase strand transfer inhibitor (INSTI) and globally one of the most frequently used antiretrovirals. DTG-based regimens have been shown to be non-inferior to efavirenz-based regimens in terms of viral suppression, and to have advantages in safety, tolerability, and ease of use.^1–3^ Furthermore, DTG’s high barrier to resistance may help preserve treatment options in patients who experience antiretroviral therapy (ART) failure.^4^ Consequently, the WHO recommended DTG-based regimen in 2019 as first-line regimen for most people living with HIV (PWH) and as second-line in people with virological failure on non-DTG-based regimens.^5^ Since then, global scale-up efforts have led to its widespread adoption in national treatment guidelines as the preferred first-line and second-line treatment for all PWH.^6^

Despite DTG’s high genetic barrier to resistance, recent cohort studies and national surveys have reported emerging resistance.^7–9^ The reported prevalence of DTG resistance among people with viraemia on DTG-based regimens is highly variable across studies and the incidence of virological failure on DTG-based ART is low.^8–11^ Resistance was typically associated with well-characterized drug resistance mutations (DRMs) in the HIV-1 integrase gene, such as R263K, G118R, N155H, and Q148H/R/K,^12^ which were classified as major INSTI DRMs by the Stanford HIVdb algorithm^13^ and by the IAS-USA.^14^ Phenotypically, R263K and N155H caused a modest, about 2-fold reduction in DTG susceptibility, G118R a strong, 19-fold reduction, whereas Q148H/R/K had only minimal effect unless combined with other resistance mutations.^12^

Previous studies have identified some integrase mutations, such as L74I, M50I, and K156N, that may also influence viral replication capacity and contribute to resistance^15,16^ yet their role in the absence of major DRMs seems rather limited.^17^ It is unclear whether these mutations reduce DTG susceptibility on their own, act synergistically with major DRMs, or contribute to the evolution of high-level resistance by enhancing replication fitness. Identifying other mutations is therefore crucial for understanding the evolutionary pathways of HIV-1 and its capacity to adapt under drug pressure from INSTIs.

In this study, we identified integrase mutations associated with predicted DTG resistance that are not currently classified as major or accessory DRMs in the Stanford HIVdb algorithm.^13^ We analysed data from the DTG RESIST study,^18^ a large international cross-sectional study investigating DTG resistance in individuals experiencing virological failure on DTG-based ART. By characterizing these mutations in a diverse clinical population, all of whom were infected with non-B subtypes, we aim to provide new insights into the evolutionary mechanisms underlying DTG resistance.

## Methods

### Ethics

Ethics approval was received by the University of Bern from the Cantonal Ethics Committee of Bern (Ref. No: 2021-01504), by the University of Kwazulu-Natal, South Africa (Ref No: BREC/00005146/2022), and all participating sites.

### Study setting

The DTG RESIST study (NCT06285110) is a large multicentre cross-sectional study^18^ nested within the International epidemiology Databases to Evaluate AIDS (IeDEA).^19^ From June 2022 to May 2025, it enrolled adults and adolescents living with HIV in Africa, Asia, and Latin America who experienced virological failure while receiving DTG-based ART.^18^ Participants were eligible if they had been on DTG-based ART for at least three months and had at least one routine viral load measurement above 1000 copies/mL. Participants were enrolled a median of 35 days (interquartile range: 20–69) after the latest routine viral load measurement >1000 copies/mL. All participants provided informed consent. Study teams collected demographic and clinical data, and whole blood samples for plasma and/or dried blood spot (DBS) preparation. The African samples were shipped to the KwaZulu-Natal Research Innovation and Sequencing Platform in Durban, South Africa. We performed sequencing if the viral load on the enrolment sample was above 1000 copies/mL.

### Sequencing and participant characteristics

Sanger sequencing was performed on the plasma or DBS samples using the HIV-1 Genotyping kit with Integrase (ThermoFisher Scientific Inc., Waltham, MA, USA), and all sequences covered the entire integrase gene (288 amino acids). Sequences were interpreted using the Stanford HIVdb algorithm (version 9.8).^13^ Nucleotide mutations that result in amino acid substitutions conferring resistance are referred to as drug resistance mutations (DRMs) according to the Stanford HIVdb algorithm. Substitutions not currently classified as major or accessory DRMs by the Stanford HIVdb algorithm are referred to as other integrase mutations. We categorized mutations in the integrase region as major DRMs for INSTIs, accessory DRMs for INSTIs, or other mutations based on the Stanford HIVdb algorithm (Table S1, available as Supplementary data at *JAC* Online). We used Rega (version 3.47)^20^ and COMET (version 2.4)^21^ to assign HIV-1 subtypes, prioritizing classification based on the integrase region. If Rega failed to assign a subtype from integrase, subtyping was based on the protease and reverse transcriptase regions using Rega; if no subtype could be assigned with Rega, we used the COMET classification for the integrase region. Only sequences from Africa were included, and for participants enrolled more than once in the study, only the sequence from the first enrolment was used in the analysis.

We compared sequence and participant characteristics with and without DTG resistance using Welch’s two-sample *t*-test to compare discrete variables (number of major DRMs, accessory DRMs, and other mutations) and chi-squared test for categorical variables (HIV-1 subtypes and country with study sites). Welch’s two-sample *t*-test was applied as it does not assume equal variances and is more robust when sample sizes differ. DTG resistance was defined as at least intermediate level of DTG resistance predicted by the Stanford HIVdb algorithm.

### Genome-wide association study-like analysis restricted to integrase region (INT-WAS)

We performed a genome-wide association study (GWAS)^22^-like analysis restricted to integrase region, which we called INT-WAS, to identify other integrase mutations statistically associated with sequences classified as resistant to DTG according to the Stanford HIVdb algorithm. We focused on the integrase region as the aim of this study was to investigate mutations in integrase region directly affecting DTG activity. After sequence alignment, a genetic distance matrix was constructed and analysed by principal coordinates analysis (PCoA).^23^ We included the top ten principal components (PCs) from PCoA as covariates in the INT-WAS model to account for population structure, which is standard in GWAS-type analyses.^24^ This adjustment helps reduce false-positive associations driven by lineage effects. INT-WAS analysis was performed using Firth’s logistic regression as the outcome was binary and the dataset included rare events and complete separation. The Firth’s method applies a penalized likelihood correction which reduces bias and enables convergence in cases of rare events or complete separation, where standard logistic regression fails.^25^ The analysis was restricted to mutations observed more than three times. We controlled multiple testing using the Benjamini–Hochberg (BH) procedure, with statistical significance defined as an adjusted *P*-value of <0.05.^26^

An additional INT-WAS analysis identified other integrase mutations enriched among HIV sequences from individuals with viremia during DTG-based treatment. We used sequences from the DTG RESIST, comprising individuals who experienced virological failure under DTG-based treatment, and drug-naïve sequences from the Los Alamos HIV-1 database, retrieved on 25 August 2025.^27^ The subtyping for the sequences from the Los Alamos database was performed using the same method as for the sequences from the DTG RESIST to ensure consistency (Table S2). To match the subtype distribution, we excluded subtype B sequences from the Los Alamos dataset as sequences from the DTG RESIST did not contain subtype B. Pairwise genetic distances were calculated to construct a distance matrix between all sequences. Based on genetic distances, each sequence from the DTG RESIST was matched to three distinct drug-naïve control sequences from the Los Alamos database with the smallest genetic distances (i.e. most genetically similar). This matching was performed using the Hungarian algorithm,^28^ an optimization method that finds the best sequence pairing to minimize overall genetic variability between datasets. As in the previous INT-WAS analysis conducted to identify other mutations associated with resistance, we considered only mutations observed at least three times in the dataset. We applied Firth’s logistic regression, adjusting for the population structure, and used the same BH correction and significance threshold (adjusted *P*-value < 0.05) to identify overrepresented mutations in sequences from viraemic individuals under DTG exposure.

### Post-INT-WAS exploratory analyses

To further quantify the impact of the identified other integrase mutations on predicted DTG resistance, we applied two complementary approaches: Fisher’s exact test and the Boruta feature-selection algorithm.^29^ Fisher’s exact test was used to identify other integrase mutations overrepresented in sequences with predicted DTG resistance compared to those without, with statistical significance defined as a Bonferroni-adjusted *P* < 0.05. The Boruta algorithm was used to identify mutations relevant to predicted DTG resistance by comparing their importance in a random forest model with that of randomly permuted mutations representing noise, and retaining only those mutations that consistently showed greater importance.^29^ We also examined the co-occurrence of identified integrase mutations in sequences defined to have DTG resistance using the pairwise Phi coefficient.

We employed ridge-penalized Bayesian logistic regression models to assess the direction of the associations (positive versus negative) between the identified mutations and genotypic DTG resistance, using three models: (i) an unadjusted model, (ii) a model adjusted for population structure by including the first ten PCs derived from the genetic distance matrix from the INT-WAS, and (iii) a model adjusted for population structure and additional demographic and clinical variables including HIV-1 subtype, country of origin, sex, number of accessory INSTI DRMs, time on DTG-based treatment, and documented exposure to first-generation INSTIs. These associations were also examined when sequences were stratified by HIV-1 subtypes. Using Fisher’s exact test, we further assessed the distribution of the identified mutations across signature DTG DRMs comprising different mutational pathways. These pathways represent distinct evolutionary trajectories by which HIV-1 acquire mutations in the integrase gene that reduce DTG susceptibility. We compared four mutually exclusive mutational pathways at four amino acid positions including G118R, Q148H/R/K, N155H, and R263K.^12^

## Results

### Study population

We included a total of 382 HIV-1 sequences from individuals experiencing viraemia while receiving DTG-based ART. Subtype C (*n* = 218, 57.1%) was the most prevalent subtype, followed by subtypes G or CRF 02_AG (*n* = 65, 17.0%), and A1 (*n* = 56, 14.7%). 104 sequences (27.2%) were defined to have intermediate or high-level resistance to DTG. Sequences with predicted DTG resistance had a median of 3 (range: 1–5) major and 1 (range: 0–3) accessory INSTI DRM. The median number of other integrase mutations was higher in sequences with predicted DTG resistance (20, range: 12–28) compared to those without (18, range: 9–31) (Table 1, Figure S1). Six out of 382 participants included in the study had documented exposure to raltegravir, a first-generation INSTI.

**Table 1.** Description of sequences with DTG resistance and without DTG resistance was defined as sequences with at least intermediate resistance to DTG predicted by the Stanford HIVdb algorithm, based on resistance-associated mutations detected in integrase region.

	DTG susceptible (*n* = 278)	DTG resistant (*n* = 104)	*P*-value
Number of major INSTI DRMs			<0.001
Mean (SD)	0.0 (0.1)	2.7 (1.0)	
Median [Min, Max]	0 [0, 1.0]	3.0 [1.0, 5.0]	
Number of accessory INSTI DRMs			<0.001
Mean (SD)	0.1 (0.4)	0.9 (0.8)	
Median [Min, Max]	0 [0, 2.0]	1.0 [0, 3.0]	
Number of other mutations on integrase			<0.001
Mean (SD)	17.9 (2.9)	20.0 (3.1)	
Median [Min, Max]	18.0 [9.0, 31.0]	20.0 [12.0, 28.0]	
HIV-1 Subtype			0.13
A1	40 (14.4%)	16 (15.4%)	
C	166 (59.7%)	52 (50.0%)	
CRF 02_AG	4 (1.4%)	1 (1.0%)	
D	9 (3.2%)	6 (5.8%)	
F	0 (0%)	2 (1.9%)	
G	38 (13.7%)	22 (21.2%)	
H	4 (1.4%)	0 (0%)	
Recombinant^a^	9 (3.2%)	3 (2.9%)	
Unknown	8 (2.9%)	2 (1.9%)	
Country (study sites)^b^			0.0467
Burkina Faso (Bobo-Dioulasso)	18 (6.5%)	2 (1.9%)	
Cameroon (Limbe)	6 (2.2%)	3 (2.9%)	
Cameroon (Yaounde)	9 (3.2%)	1 (1.0%)	
Congo (CTA Brazzaville)	13 (4.7%)	4 (3.8%)	
Congo (CTA Pointe-Noire)	15 (5.4%)	10 (9.6%)	
Cote d'Ivoire (CePReF)	6 (2.2%)	8 (7.7%)	
Cote d'Ivoire (CNTS)	12 (4.3%)	6 (5.8%)	
Kenya (Eldoret)	8 (2.9%)	7 (6.7%)	
Malawi (Lighthouse)	17 (6.1%)	6 (5.8%)	
Malawi (MPC)	44 (15.8%)	12 (11.5%)	
Rwanda (Kicukiro)	2 (0.7%)	0 (0%)	
Uganda (Masaka)	12 (4.3%)	8 (7.7%)	
Uganda (Mbarara)	14 (5.0%)	2 (1.9%)	
Zambia (CIDRZ)	70 (25.2%)	19 (18.3%)	
Zimbabwe (Newlands)	32 (11.5%)	16 (15.4%)	

P-value was calculated using a two-sample t-test for numerical variables (number of major DRMs, accessory DRMs, and other mutations) and a chi-squared t-test for categorical variables (HIV-1 subtypes and country with study sites). DTG = dolutegravir. INSTI = integrase strand transfer inhibitor. DRM = drug resistance mutation.

^a^Recombinant includes CRF 04_CPX (n = 1), CRF 06_CPX (n = 8), CRF 25_CPX (n = 1), CRF 11_CPX (n = 1), CRF 09_CPX (n = 1).

^b^CTA = clinical treatment centres. CePReF = Centre de Recherche et de Prévention des Maladies Infectieuses. CNTS = Centre National de Transfusion Sanguine. MPC = Martin Preuss Centre. CIDRZ = Centre for Infectious Disease Research in Zambia.

### Association of other integrase mutations with predicted DTG resistance

**Figure 1.**
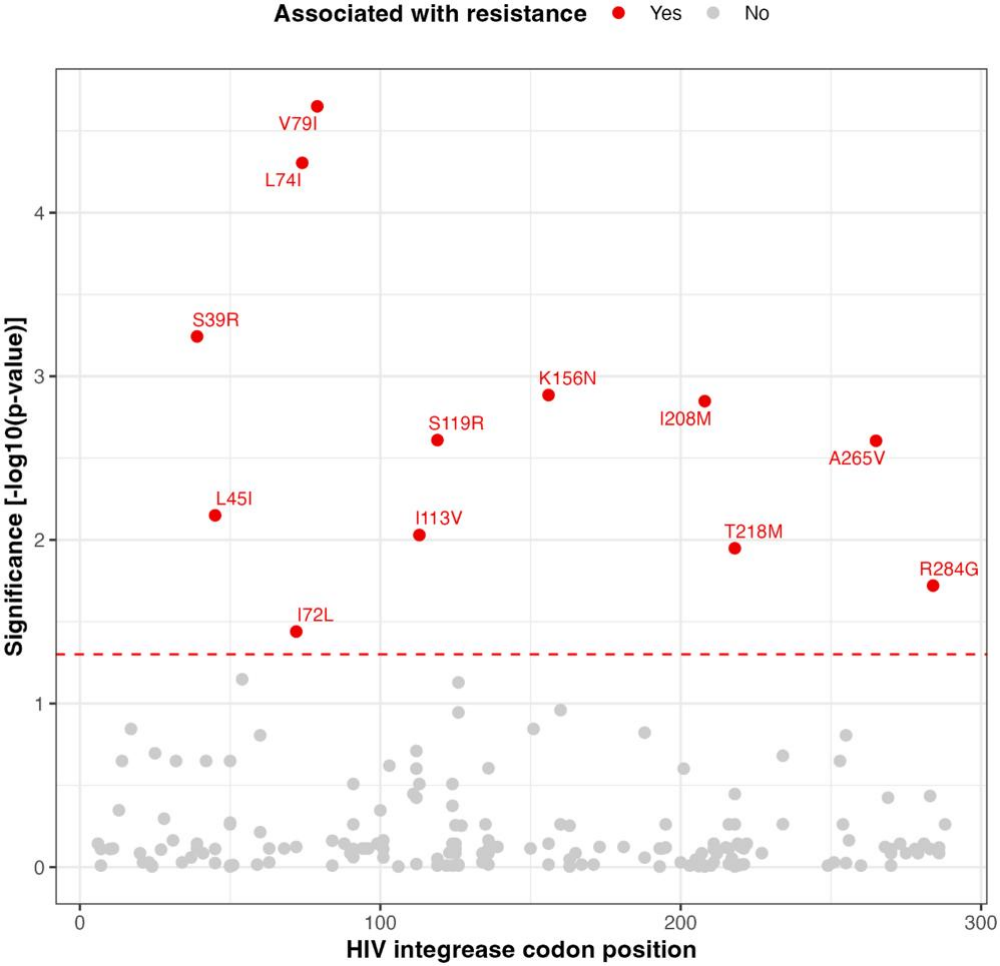
Genome-wide association study-like analysis restricted to integrase region (INT-WAS) of other integrase mutations and associations with genotypic DTG resistance. For INT-WAS analysis, Firth’s logistic regression was applied, including the first 10 PCs to account for population structure. *P*-values were adjusted using the BH procedure, with a significance threshold of adjusted *P*-value < 0.05. The name of significant mutations were displayed and mutations identified in the INT-WAS analysis for association with DTG resistance (Figure 1) are highlighted in red. DTG = dolutegravir; PC = principal component; BH = Benjamini–Hochberg.

We identified 12 other integrase mutations (i.e. not currently classified as major or accessory INSTI DRMs in the Stanford HIVdb) significantly associated with predicted DTG resistance using the INT-WAS approach. These mutations comprise S39R, L45I, I72L, L74I, V79I, I113V, S119R, K156N, I208M, T218M, A265V, and R284G (Figure 1). The frequency of these identified mutations in INSTI-naïve individuals, based on data from the Stanford HIVdb, ranged from 0% to 15.2% (Table S3). When stratified by subtype, some mutations exhibited markedly higher prevalence within specific subtypes (Table S3). When comparing prevalence of other integrase mutations in sequences with Fisher’s exact test, 23 mutations including seven (S39R, I72L, V79I, K156N, I208M, T218M, and A265V) of the 12 identified mutations were significantly overrepresented among sequences with predicted DTG resistance compared to those without (Table S4). For four (S39R, I72L, V79I, and T218M) of the seven overrepresented identified mutations, odds ratios were infinite because they were absent in sequences without DTG resistance. Of the 12 mutations identified in the INT-WAS analysis, eight (S39R, L45I, I72L, V79I, K156N, I208M, T218M, and A265V) were also included in the list of mutations considered predictive of DTG resistance by the Boruta feature-selection algorithm (Figure S2). Finally, two mutation pairs (I72L-K156N and V79I-R284G) from the 12 identified mutations showed significant pairwise associations (Figure S3). Both associations were weakly positive with Phi coefficients of 0.31 and 0.28, respectively.

### Adjusted analyses and mutational pathways

**Figure 2.**
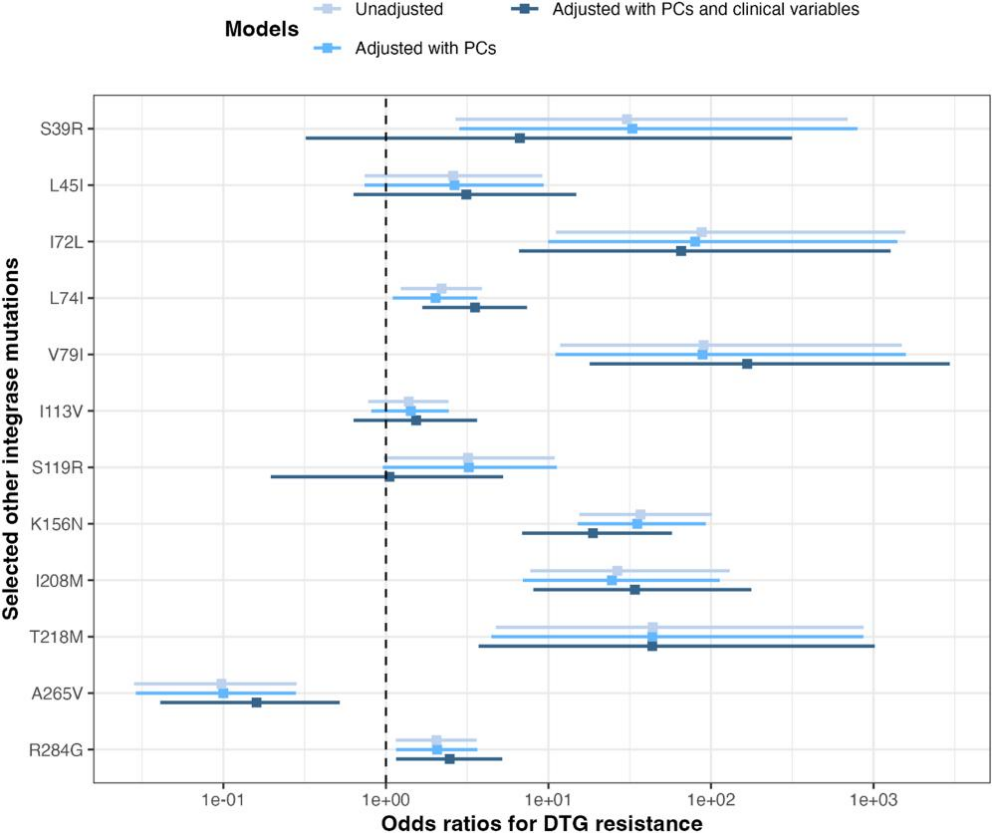
Odds ratios for DTG resistance. Odds ratios with 95% credible intervals were calculated using a Bayesian regression model. Mutations identified from the INT-WAS Firth’s regression were included in the analysis. Different models are in different colours. Unadjusted model included each other integrase mutation without adjusting for any other variables. Different covariates were added into different models: (i) adjusted model with PCs include the first ten PCs used in the INT-WAS Firth’s regression to adjust for population structure, (ii) adjusted model with PCs and clinical variables include the first 10 PCs and demographic and clinical variables including HIV-1 subtype, country, sex, number of accessory INSTI DRMs, time on DTG-based treatment, and documented exposure to first-generation INSTI. DTG = dolutegravir. INT-WAS = genome-wide association study-like analysis restricted to integrase region. PC = principal component. INSTI = integrase strand transfer inhibitor. DRMs = drug resistance mutations.

In the Bayesian logistic regression models adjusting for population structure (i.e. included PCs from the INT-WAS analysis) and demographic and clinical variables, seven (I72L, L74I, V79I, K156N, I208M, T218M, and R284G) of the 12 mutations were significantly positively associated with predicted DTG resistance, whereas one (A265V) showed a significant negative association (Figure 2). The V79I mutation showed the strongest positive association with an adjusted odds ratio (aOR) of 167.1 (95% credible intervals (Crl) = 17.9–2947.6), followed by I72L (aOR = 65.6, 95% Crl = 6.6–1273.7).

**Figure 3.**
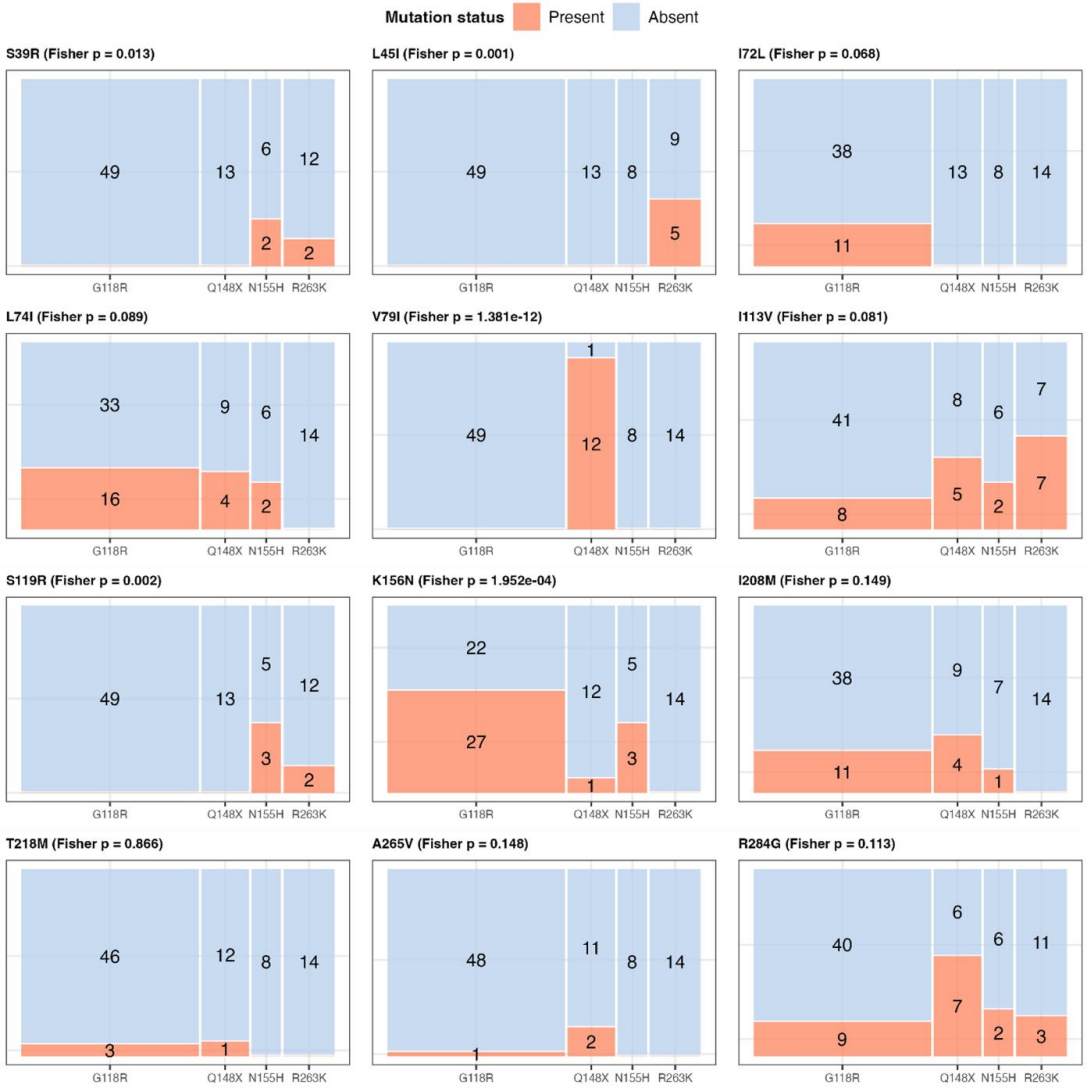
Distribution of 12 selected integrase mutations across INSTI resistance pathways. Fisher’s exact test was performed for each identified mutation across four major INSTI resistance pathways (G118R, Q148X, N155H, R263K) in sequences with DTG resistance. Q148X refers to Q148H/K/R pathways. Sequences with each mutually exclusive pathway were included (*n* = 84), while those with more than two mixed pathways were excluded (*n* = 20). Mutation status is categorized as absent (blue) and present (red), and the size of each rectangle is proportional to the number of sequences in each category. The number of sequences in each rectangle is shown, except when it is 0. Panel titles display the mutation name and the *P*-value from Fisher’s exact test, indicating whether the distribution differs significantly among pathways. INSTI = integrase strand transfer inhibitor. DTG = dolutegravir.

Most associations between mutations and predicted DTG resistance were consistent across HIV-1 subtypes (Figure S4). However, comparisons were limited due to small sample sizes in subtypes A1 and G or AG. Five of the 12 mutations (S39R, L45I, V79I, S119R, and K156N) were associated with specific INSTI resistance pathways (Figure 3). Particularly, for the V79I mutation, all DTG resistant cases (*n* = 12) occurred with the Q148H/R/K pathway. Similarly, for the L45I mutation, all five resistant cases occurred with R263K.

### Comparison with drug-naïve sequences

For this analysis, we included 1528 sequences in total (382 sequences from the DTG RESIST and 1146 drug-naïve controls from the Los Alamos database). The comparison with the drug-naïve sequences showed that 25 other integrase mutations (S24D, N27H, V32I, K42Q, M50I/R, V54I, S57G, I60V, I72L, L74I, V79I, Q95P, T124A, G134N, I135L, K156N, D167E, I182V, I208M, T218I, I251L, D278G, S283D, and R284G) were overrepresented in sequences from viraemic individuals under DTG-based treatment (Figure S5). Of these, six mutations (I72L, L74I, V79I, K156N, I208M, and R284G) had been identified as associated with genotypic DTG resistance in the previous INT-WAS analysis. The other 19 mutations, although overrepresented in sequences from DTG-exposed viraemic individuals, were not enriched in those exhibiting predicted DTG resistance.

## Discussion

In this study, we identified 12 integrase mutations that are not classified by Stanford HIVdb as major or accessory INSTI DRMs but are strongly associated with predicted DTG resistance in this dataset of non-B subtype viruses. Most of these mutations were consistently overrepresented in sequences with predicted DTG resistance compared to ones without. Some mutations, including V151, were overrepresented in sequences with predicted DTG resistance but were not identified as associated with genotypic DTG resistance in the INT-WAS analysis. This likely reflects confounding by subtype or population structure. Notably, several of these mutations also showed high prevalence among INSTI-naïve individuals, particularly in certain subtypes, suggesting that some observed associations may reflect natural subtype-specific polymorphisms. However, this does not preclude their potential contribution to genotypic DTG resistance, as such polymorphisms may persist under drug pressure due to their low fitness cost. Their associations with resistance persisted after adjusting for demographic and clinical factors and were robust across HIV-1 subtypes. Notably, five of the 12 mutations were linked to specific DTG mutational pathway defined by their specific signature DTG resistance mutations, and six were enriched in sequences from DTG-exposed viraemic individuals, suggesting a potential role in sustaining viral replication under drug pressure.

Several of the 12 mutations have previously been proposed as compensatory. For example, the K156N mutation has been characterized *in vitro* as a potential accessory mutation that enhances DTG resistance when co-occurring with a major INSTI DRM N155H.^16^ In line with this, our study found that the K156N mutation is also associated with other signature major INSTI DRMs, particularly G118R, indicating that its potential role is not limited to the N155H pathway but may instead represent a more general compensatory mutation. Additionally, L74I has been recognized as a signature mutation in HIV-1 subtype A6, where it may restore replication fitness in the presence of major INSTI DRMs, particularly in the context of the long-acting INSTI cabotegravir.^14^ Although the A265V mutation is known to occur naturally as a polymorphism across different HIV-1 subtypes,^13,30^ our study found it to be negatively associated with predicted DTG resistance. Further work is required to understand whether and how its presence, in the absence of a compensatory role, might interfere with resistance development.

The majority of the identified mutations showed no significant pairwise associations, suggesting they tend to emerge independently in sequences with predicted DTG resistance, unlike the structured pathways observed with major INSTI DRMs.^8,12^ Nonetheless, five of the 12 mutations were linked to established INSTI resistance pathways, indicating that while they may arise independently, they could still converge on key mechanisms of DTG resistance. To date, no study has systematically examined the impact of combinations of other mutations on resistance dynamics. An analysis of the Italian ICONA cohort reported that the most frequently observed pattern among individuals experiencing virological failure involved the L74I mutation.^15^ However, only a few mutation combinations were detected, underscoring that our understanding of potential synergistic effects among these mutations in shaping resistance and viral fitness is limited.

Interestingly, we observed that while some other integrase mutations were associated with predicted DTG resistance and enriched in sequences from viraemic individuals (I72L, L74I, V79I, K156N, I208M, and R284G), others were linked exclusively to one of these outcomes. Although the interpretation of their impact on the viral fitness landscape would be limited based on our findings, these distinct groups of mutations may reflect different resistance mechanisms in HIV-1, suggesting that resistance and viral replication, while interconnected, do not always overlap.

A strength of our study is the inclusion of, to the best of our knowledge, the largest dataset of non-B HIV-1 subtype sequences of PWH with virological failure on DTG-based ART. Such sequences have been underrepresented in previous studies. This is particularly relevant given prior reports of the different prevalence of other integrase mutations between subtype B and non-B viruses.^31,32^ However, our findings rely exclusively on genotypic resistance data, without accompanying phenotypic resistance, drug-level measurements or robust adherence data. Such data would help to clarify the impact of the identified mutations on viral fitness and resistance dynamics. Additional study limitations include the cross-sectional design, sequencing limited to viral loads above 1000 copies/mL, and the absence of pre-DTG-based regimen sequences. The lack of baseline sequences restricted our ability to capture early mutational events in DTG resistance and assess the influence of background resistance, such as nucleoside reverse transcriptase inhibitor-associated mutations, on DTG resistance. It also constrained our assessment of the selective pressures driving resistance, as some mutations may have reverted over time in the presence of persistent viraemia. Lastly, we did not perform an additional phylogenetic analysis for subtype confirmation or recombination beyond the subtyping procedures applied. However, the Rega subtyping tool does rely on phylogenetic methods for subtype assignment, and recombination identified by these tools was relatively uncommon in our dataset (12 out of 382 sequences). In addition, to further mitigate potential bias from genetic diversity, we included principal components derived from pairwise genetic distances as a covariate in our analysis.

In conclusion, our analysis suggests that several other integrase mutations may be associated with predicted genotypic DTG resistance and viraemia during DTG-based treatment. Although their effects on viral fitness remain uncertain, they could represent compensatory mutations reducing the fitness cost of major INSTI DRMs. Phenotypic resistance testing may validate their functional roles and contributions to resistance dynamics.

## Supplementary Material

dkag088_Supplementary_Data

## Data Availability

The sequence data are available in GenBank under accession numbers PV008448-PV008617, PV419540-PV419544, PV419550, PV419551, PV419554, PV419555, PV419557-PV419583, PV467483-PV467495, PV816612-PV816623, PV816625, PV816629-PV816633, PV816635-PV816650, PV816652-PV816682, PV816684, PV816687, PV816689-PV816692.

## Transparency declarations

H. F. G. has received grants from the Swiss National Science Foundation, the Swiss HIV Cohort Study, the Yvonne Jacob Foundation, University of Zurich's Clinical Research Priority Program, Zurich Primary HIV Infection, Systems. X, the Bill & Melinda Gates Foundation, the US NIH, Gilead Sciences, ViiV Healthcare, and Roche; has received honoraria for advisory boards from Gilead Sciences, ViiV Healthcare, Janssen, GSK, Johnson & Johnson, and Novartis; has received honoraria for a data safety monitoring board from Merck and has received a travel grant from Gilead Sciences. R. L. has received financial support (payment to the institution) for the present work from the NIH NIAID and has received grants (payment to institution) from the NIH NIAID for an unrelated project. R. D. K. receives grants from the Swiss National Science Foundation, the US NIH, and Gilead Sciences. All other authors: none to declare.

## Supplementary data

Figures S1 to S5 and Tables S1 to S4 are available as Supplementary data at *JAC* Online.
