# Supplementary material for: Novel integrase mutations linked to genotypic DTG resistance in African non-B HIV-1 strains: the DTG RESIST study
Source: J Antimicrob Chemother. 2026 Mar 24;81(4):dkag088. doi: 10.1093/jac/dkag088 (PMC13008830; doi:10.1093/jac/dkag088)
Supplement: dkag088_Supplementary_Data [file dkag088_supplementary_data.docx]

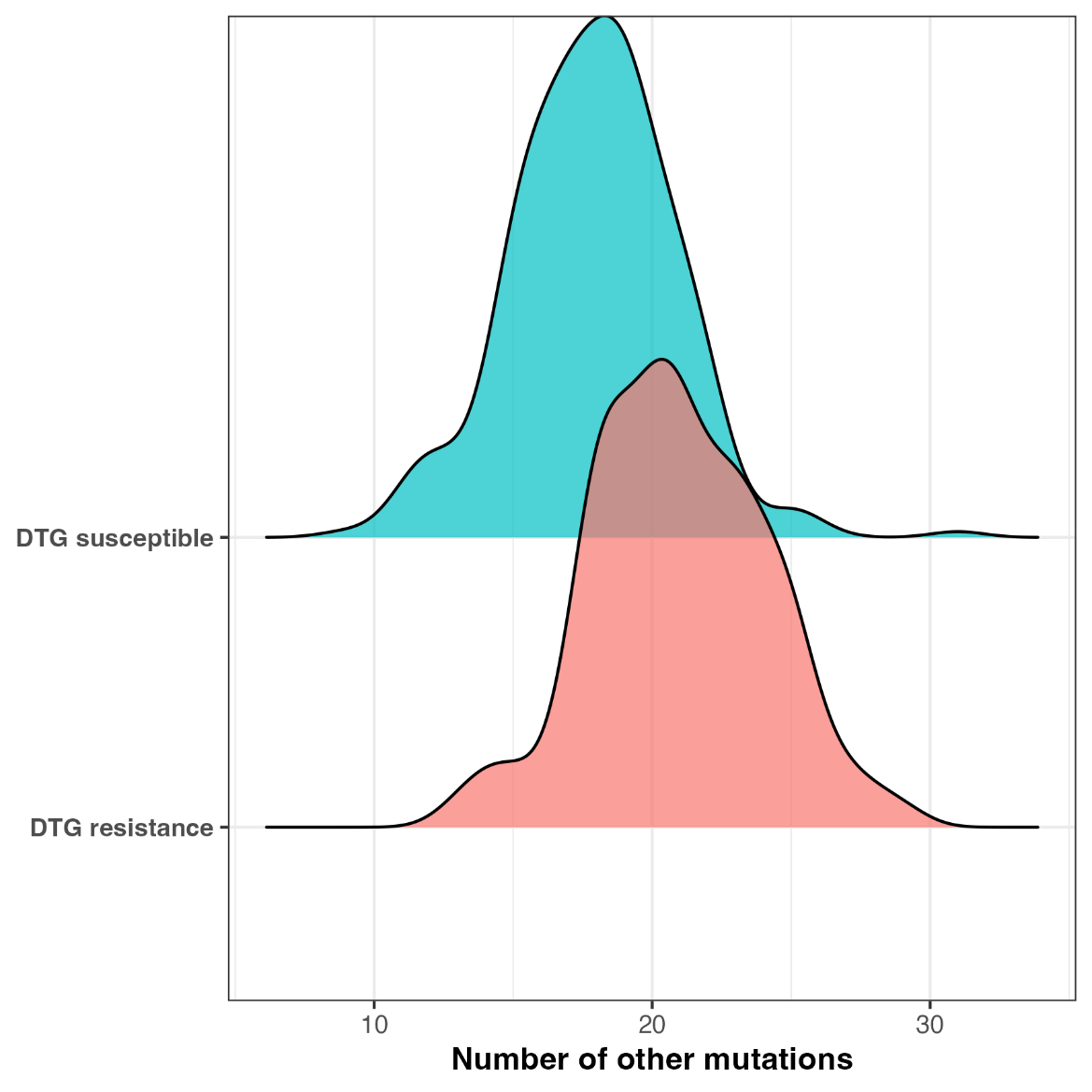


**Supplementary figure 1 Density of number of other integrase mutations from sequences with DTG resistance (red) and sequences without DTG resistance (blue).** Welch two sample t-test was performed to compare the mean number of other mutations between sequences with predicted DTG resistance (“DTG resistance”) and those without (“DTG susceptible”): t-statistics = -8.75 [95% confidence intervals: -3.78 to -2.39], p-value = 1.735e-15. DTG = dolutegravir.

**
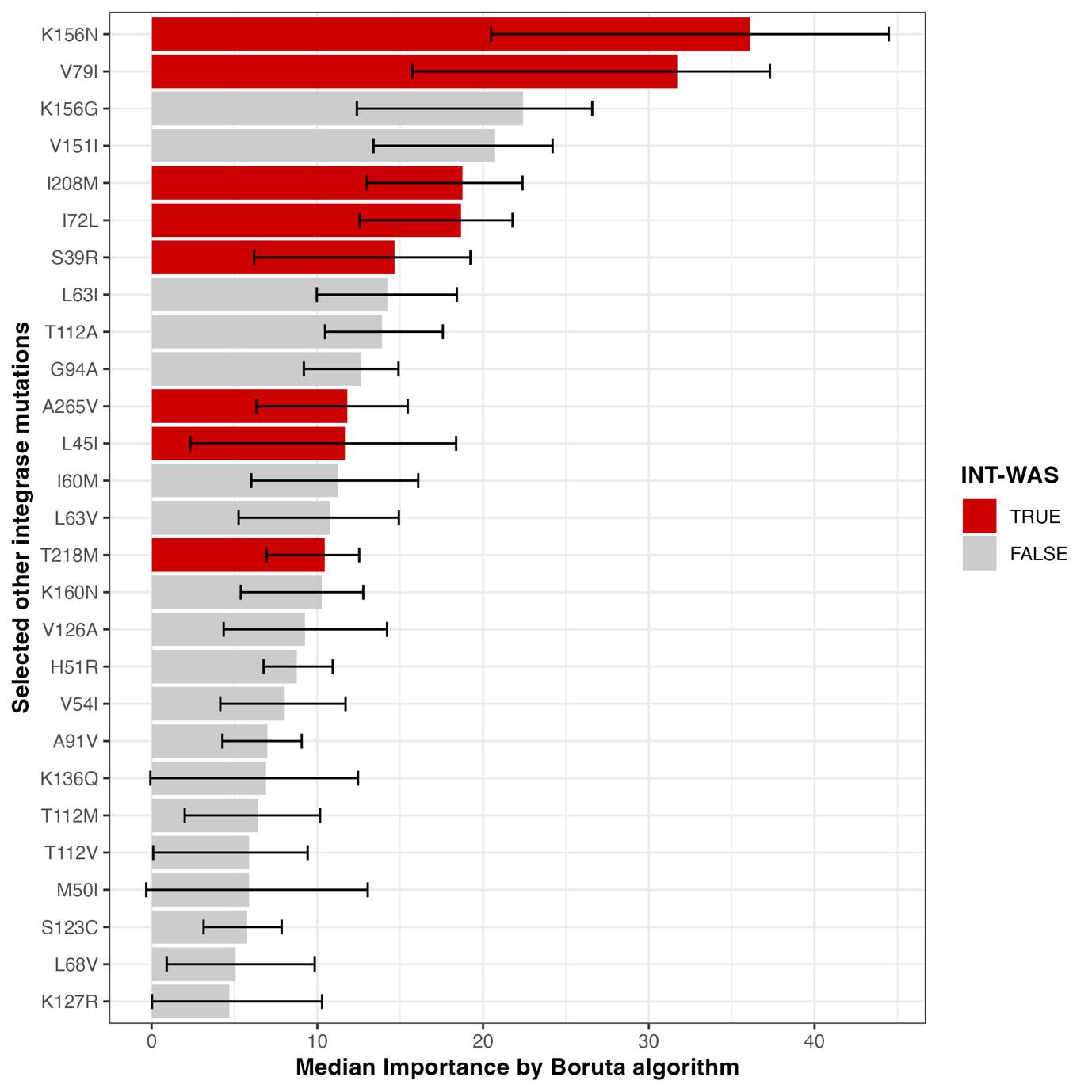
Supplementary Figure 2 Median importance scores for integrase mutations calculated using the Boruta algorithm.** The Boruta algorithm identifies features relevant for predicting DTG resistance. Mutations classified as confirmed by the Boruta algorithms are listed, representing mutations which importance scores were significantly above permuted mutations. Higher median importance scores indicate greater predictive relevance. If they have been identified by the INT-WAS analysis to be associated with predicted DTG resistance, they are coloured by red. DTG = dolutegravir. INT-WAS = genome-wide association study-like analysis restricted to integrase region.

**
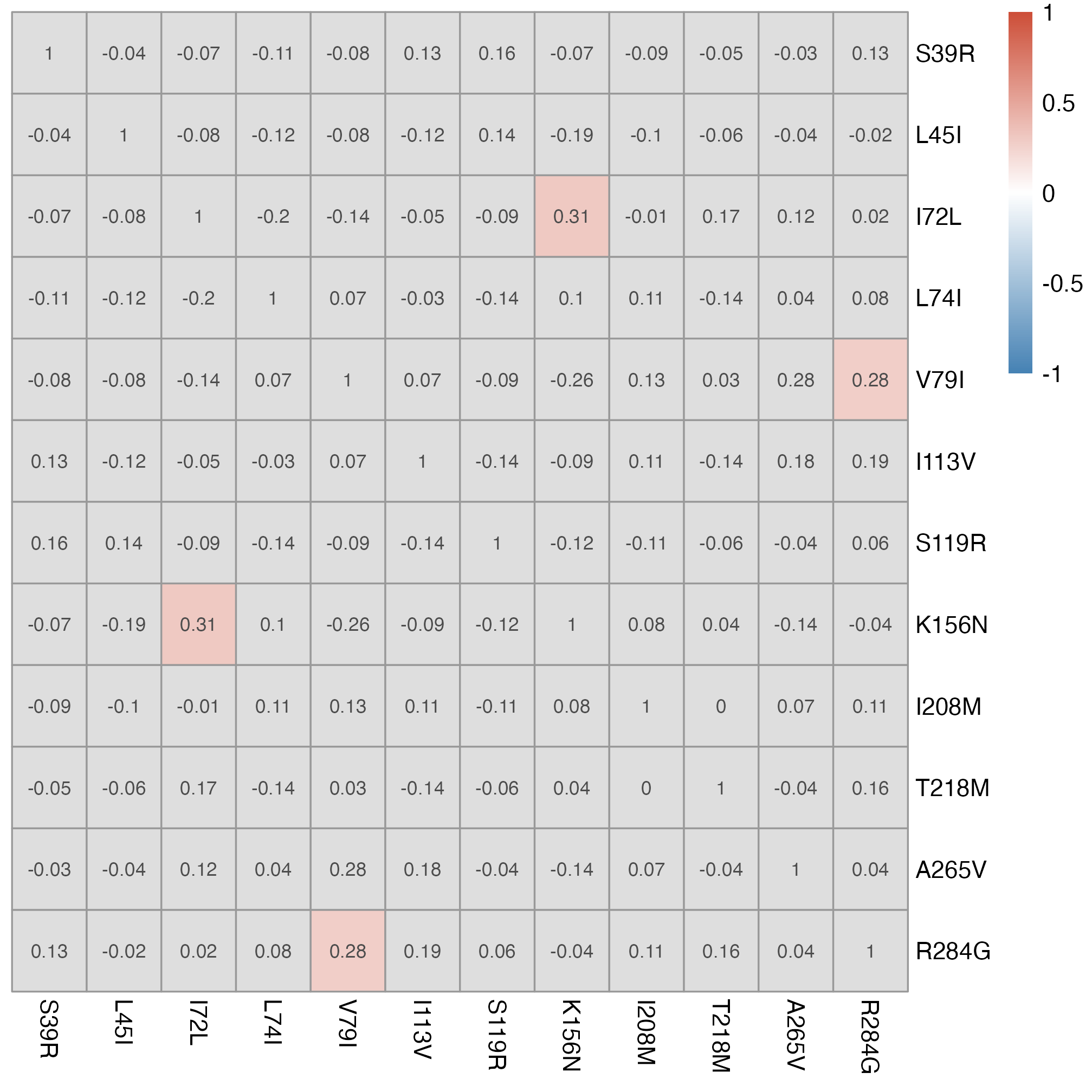
Supplementary Figure 3 Pairwise Phi coefficients among selected mutations in sequences with DTG resistance.** The Phi coefficient (φ) quantifies the strength and direction of linear relationships between selected other integrase mutations. Other integrase mutations identified from INT-WAS Firth’s regression were included in the analysis. Non-significant associations (BH-adjusted p-value > 0.05) were marked as grey. P-values were derived from contingency table tests: Fisher’s exact test was used when cell counts were < 5, and Pearson’s chi-squared test was applied otherwise. Red indicates strong positive co-occurrence, while blue indicates negative co-occurrence (i.e., their occurrence is independent). DTG = dolutegravir. INT-WAS = genome-wide association study-like analysis restricted to integrase region. BH = Benjamini-Hochberg.

**
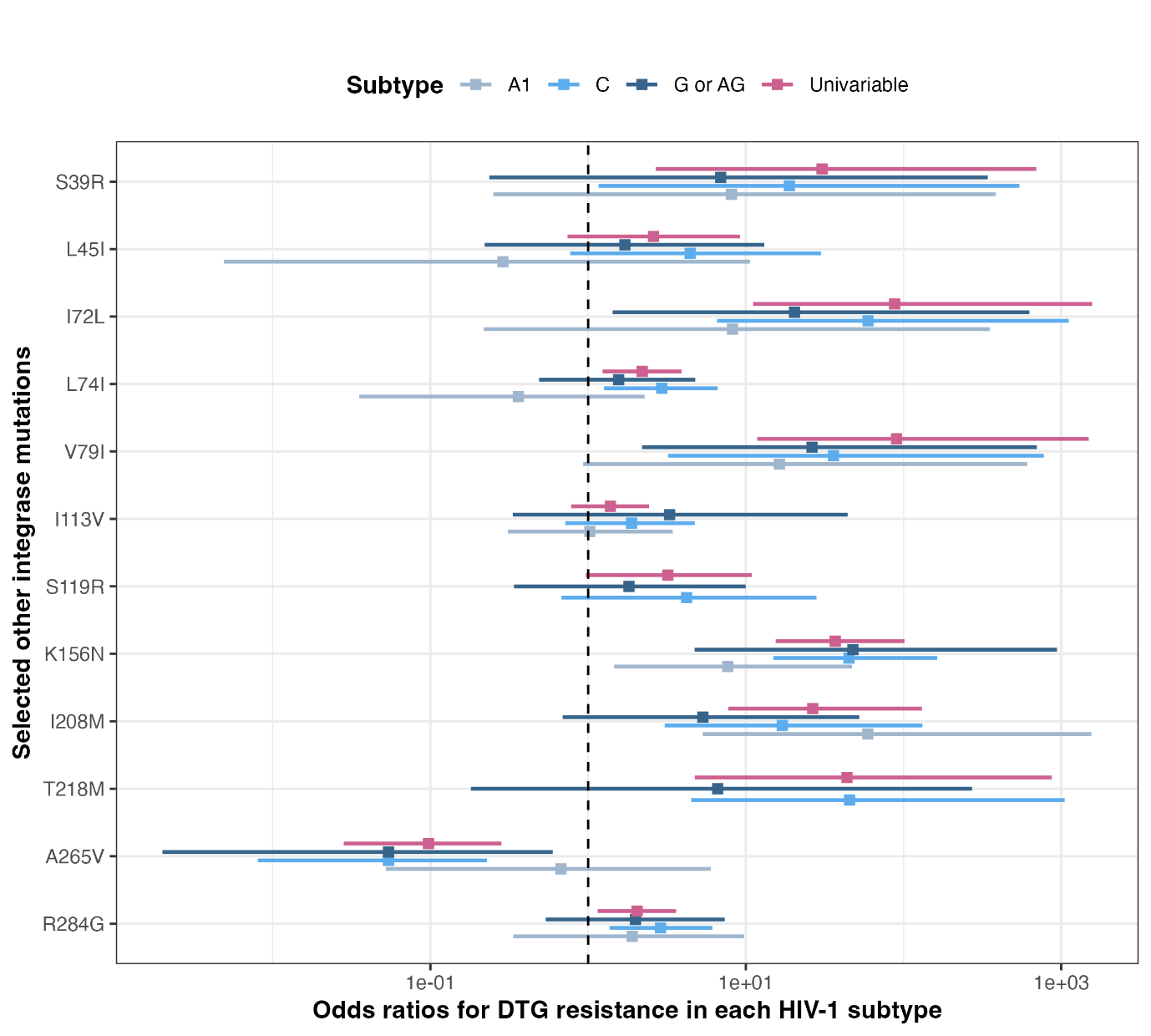
Supplementary Figure 4 Odds ratios for DTG resistance in HIV-1 subtypes.** Odds ratios with 95% credible intervals were calculated using a Bayesian regression model. Other mutations identified from INT-WAS Firth’s regression were tested within each subtype. Among them, other mutations absent in each subtype were excluded in the analysis with the respective subtype. Each model in each subtype is shown in different colours. DTG = dolutegravir. INT-WAS = genome-wide association study-like analysis restricted to integrase region.

**
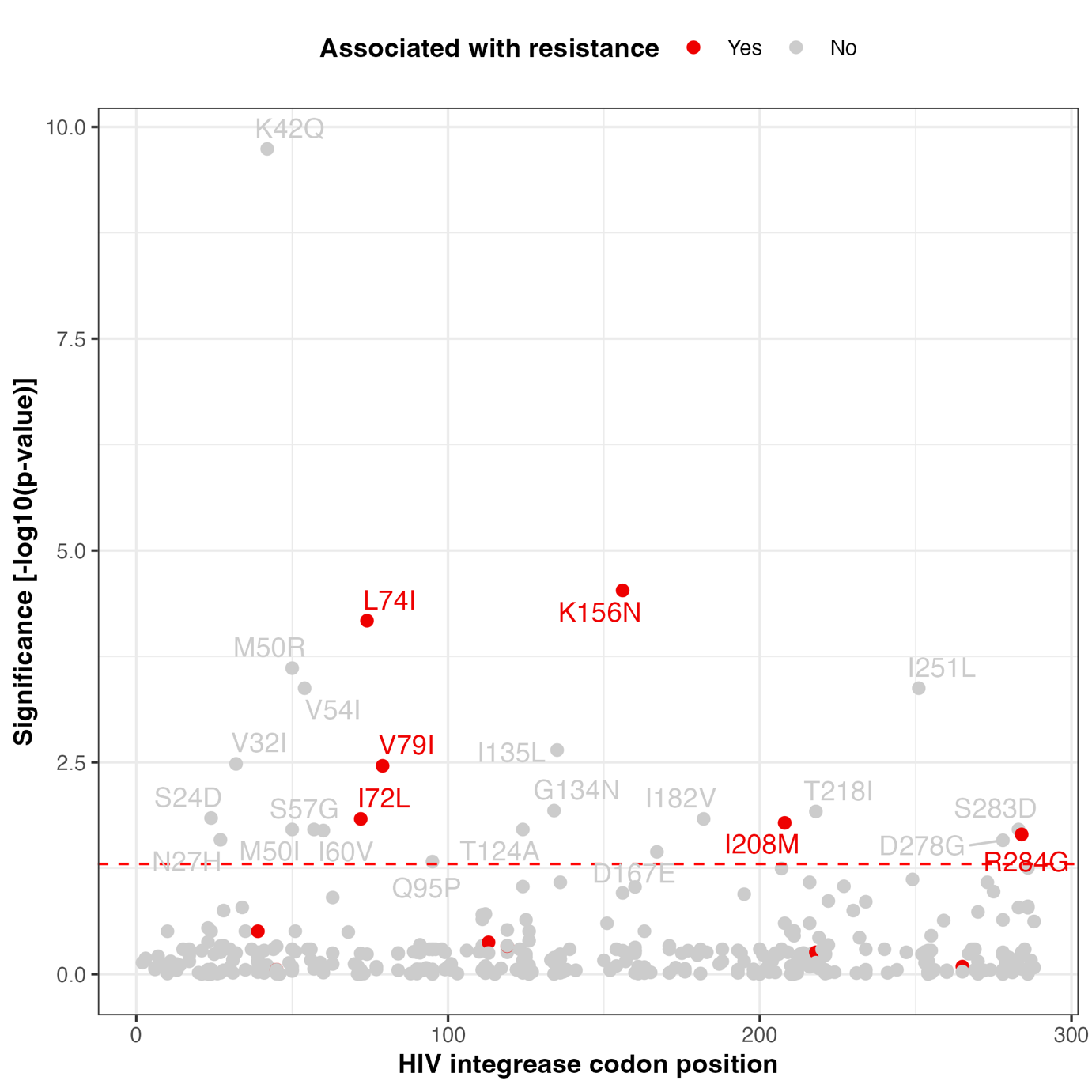
Supplementary Figure 5** **Genome-wide association study-like analysis restricted to integrase region (INT-WAS) of other integrase mutations and their associations with sequences from viraemic individuals under DTG-based treatment.** A case-control design (1:3 matching) was used to select sequences with the lowest genetic distance for the analysis. For INT-WAS, Firth’s logistic regression was applied, including the first 10 PCs to account for population structure. P-values were adjusted using the BH procedure, with a significance threshold of adjusted p-value < 0.05. The name of significant mutations were displayed and mutations identified in the INT-WAS for association with predicted DTG resistance (Figure 1) are highlighted in red. DTG = dolutegravir. PC = principal component. BH = Benjamini-Hochberg.

**Supplementary Table 1. Major and accessory drug resistance mutations included in the analysis.** Only mutations observed at least 3 times in the sequences were included.

| **Major drug**  **resistance mutations** | **Accessory drug resistance mutations** |
| --- | --- |
| T66A/I  G118R  E138A/K  G140A  S147G  Q148K/R  N155H  R263K | H51Y  L74I/M  Q95K  T97A  G149A  S153A  E157Q  G163R |

**Supplementary Table 2.** **Subtype distribution of drug-naïve sequences from the Los Alamos HIV-1 Database.** Rega (version 3.47) and COMET (version 2.4) were used to assign HIV-1 subtypes, prioritizing classification based on the integrase region. If Rega failed to assign a subtype from integrase, subtyping was based on the protease and reverse transcriptase regions using Rega; if no subtype could be assigned with Rega, we used the COMET classification for the integrase region.

| **Subtype** | **Frequency** |
| --- | --- |
| A | 2 (0.2%) |
| A1 | 168 (14.7%) |
| C | 588 (51.3%) |
| CRF 01_AE | 16 (1.4%) |
| CRF 02_AG | 43 (3.8%) |
| D | 24 (2.1%) |
| F | 10 (0.9%) |
| G | 164 (14.3%) |
| J | 3 (0.3%) |
| Rec | 91 (7.9%) |
| Unassigned | 37 (3.2%) |

**Supplementary Table 3. Prevalence of the identified mutations among INSTI-naïve individuals.** Estimates were derived from the Stanford HIVdb database (data version updated on 2025-10-07).

|  | **Prevalence in INSTI-naïve individuals (%)** | | | | | | |
| --- | --- | --- | --- | --- | --- | --- | --- |
| **Mutations** | **Overall** | **A** | **C** | **D** | **F** | **G** | **CRF02_AG** |
| S39R | 0.16 | 0.25 | 0.06 | 0 | 0.14 | 0 | 0.06 |
| L45I | 2.73 | 0.72 | 1.31 | 1.84 | 2.51 | 0.51 | 1.87 |
| I72L | 0 | 0 | 0 | 0 | 0 | 0 | 0 |
| L74I | 8.8 | 39.35 | 5.76 | 3.82 | 6.28 | 12.41 | 15.53 |
| V79I | 0.11 | 0.07 | 0.06 | 0 | 0 | 0 | 0 |
| I113V | 15.22 | 69.12 | 2.44 | 83.78 | 3.27 | 1.4 | 1.87 |
| S119R | 2.56 | 0.77 | 1.51 | 1.54 | 0.82 | 0.94 | 2.72 |
| K156N | 9.11 | 0.35 | 0.49 | 2.58 | 3.24 | 0.47 | 0.29 |
| I208M | 1.09 | 2.36 | 0.9 | 0.39 | 1.1 | 3.57 | 1.14 |
| T218M | 0.15 | 0.11 | 0.46 | 0.69 | 0 | 0 | 0.31 |
| A265V | 12.13 | 5.95 | 31.82 | 29.95 | 3.44 | 2.23 | 15.9 |
| R284G | 4.14 | 4.8 | 6.65 | 5.18 | 1.42 | 2.91 | 6.53 |

**Supplementary Table 4. Other integrase mutations associated with predicted DTG resistance.** 23 other integrase mutations are listed, each significantly overrepresented in sequences with predicted DTG resistance, using fisher’s exact test (‘Odds ratios [95% CI]’ and ‘P-value’). P-value was adjusted for multiple comparisons using the Benjamini-Hochberg procedure. Number of each mutation in sequences with and without predicted DTG resistance is described in the first two columns. DTG = dolutegravir.

| **Mutation** | **Number in sequences without DTG resistance** | **Number in sequences with DTG resistance** | **Odds ratios [95% CI]** | **P-value** | **GWAS** |
| --- | --- | --- | --- | --- | --- |
| V151I | 1 | 17 | 53.56 [8.16, 2242.48] | <0.001 | No |
| K156N | 5 | 43 | 37.95 [14.27, 128.48] | <0.001 | Yes |
| I208M | 2 | 18 | 28.61 [6.64, 257.5] | <0.001 | Yes |
| T112A | 5 | 21 | 13.7 [4.83, 47.9] | <0.001 | No |
| L63I | 5 | 21 | 13.7 [4.83, 47.9] | <0.001 | No |
| T112M | 2 | 7 | 9.89 [1.84, 99.17] | 0.019 | No |
| L63V | 3 | 8 | 7.59 [1.78, 45.36] | 0.019 | No |
| V126A | 4 | 10 | 7.24 [2.03, 32.4] | 0.006 | No |
| V54I | 4 | 9 | 6.45 [1.75, 29.35] | 0.017 | No |
| I60M | 10 | 17 | 5.21 [2.16, 13.24] | <0.001 | No |
| T218I | 105 | 20 | 0.39 [0.22, 0.69] | 0.006 | No |
| T112V | 237 | 70 | 0.36 [0.2, 0.63] | 0.002 | No |
| A265V | 65 | 3 | 0.1 [0.02, 0.31] | <0.001 | Yes |
| I72L | 0 | 12 | Inf [8.14, Inf] | <0.001 | Yes |
| V79I | 0 | 13 | Inf [9.02, Inf] | <0.001 | Yes |
| S123C | 0 | 4 | Inf [1.8, Inf] | 0.041 | No |
| T218M | 0 | 6 | Inf [3.26, Inf] | 0.005 | Yes |
| K160N | 0 | 4 | Inf [1.8, Inf] | 0.041 | No |
| G94A | 0 | 10 | Inf [6.43, Inf] | <0.001 | No |
| K156G | 0 | 11 | Inf [7.28, Inf] | <0.001 | No |
| A91V | 0 | 4 | Inf [1.8, Inf] | 0.041 | No |
| H51R | 0 | 6 | Inf [3.26, Inf] | 0.005 | No |
| S39R | 0 | 4 | Inf [1.8, Inf] | 0.041 | Yes |
